# Knowledge, Attitudes, and Social Responsiveness Toward Corona Virus Disease 2019 (COVID-19) Among Chinese Medical Students—Thoughts on Medical Education

**DOI:** 10.3389/fmed.2021.647679

**Published:** 2021-06-11

**Authors:** Haojun Yang, Yue Zheng, Fang Yu, Bohao Cheng, Ziqing Zhu, Sheng Liao, Yu Chen, Jianzhen Wu, Fang Wang

**Affiliations:** ^1^Department of Endocrinology, The Third Xiangya Hospital of Central South University, Changsha, China; ^2^Department of Neurology, Xiangya Hospital of Central South University, Changsha, China; ^3^West China School of Medicine, Sichuan University, Chengdu, China; ^4^The Third Xiangya Hospital of Central South University, Changsha, China

**Keywords:** knowledge, attitude, COVID-19, Chinese medical students, social responsiveness

## Abstract

**Purpose:** To assess knowledge, attitudes, and social responsiveness toward COVID-19 among Chinese medical students.

**Methods:** Self-administered questionnaires were used to collect data from 889 medical students in three well-known Chinese medical universities. The questionnaire was comprised of three domains which consisted of demographic characteristic collection, seven items for knowledge, and eight items for attitudes and social responsiveness toward COVID-19. Data from different universities were lumped together and were divided into different groups to compare the differences, including (1) students at the clinical learning stage (Group A) or those at the basic-medicine stage (Group B) and (2) students who have graduated and worked (Group C) or those newly enrolled (Group D).

**Results:** Medical students at group B had a weaker knowledge toward COVID-19 than did students at group A, especially in the question of clinical manifestations (*p* < 0.001). The percentage of totally correct answers of COVID-19 knowledge in group C was higher than that in Group D (*p* < 0.001). There were significant differences between groups C and D in the attitudes and social responsiveness toward COVID-19. Surprisingly, we found that the idea of newly enrolled medical students could be easily affected by interventions.

**Conclusions:** In light of this information, medical education should pay attention not only to the cultivation of professional knowledge and clinical skills but also to the positive interventions to better the comprehensive qualities including communicative abilities and empathy.

## Introduction

Widespread interruptions of medical education have been seen throughout history by major conflicts or infectious pandemic (1). The structure, delivery, and future of both undergraduate and graduate medical education suffered as a result, requiring educators and learners to adapt to learning from a distance while aiming for normalization (2, 3). The suboptimal replication of patient encounters and gauging audience understanding and identifying knowledge gaps seemed to be the direct challenges faced by educators during the pandemic (3, 4). Importantly, the pandemic sounded a wake-up call for us and posed new challenges to medical education, such as insufficient emphasis on public health emergency preparedness, unsophisticated interdisciplinary cooperation mechanism, and insufficient guidance in medical ethics (5). In other words, it put forward higher and stricter requirements for disease prevention and control in the future, especially for public health security and emergency response capacity of infectious diseases (5, 6).

In late December 2019, a novel coronavirus pneumonia (NCP) epidemic occurred in Wuhan, Hubei Province, China. The World Health Organization (WHO) named the pneumonia caused by new coronavirus as Corona Virus Disease 2019 (COVID-19), and the International Committee on Taxonomy of Viruses (ICTV) named this pathogen as Severe Acute Respiratory Syndrome Coronavirus 2 (SARS-COV-2) (7). Compared with SARS, COVID-19 has the characteristics of a long incubation period, strong infectivity, and no obvious upper respiratory symptoms (8, 9). COVID-19 has been declared as a pandemic disease by the WHO on March 11, 2020, with approximately 81,000 confirmed cases in China and over 40,000 confirmed cases in other countries cumulatively (10, 11). Therefore, the Chinese government has implemented strict public health measures against the spread of COVID-19 and dispatched medical personnel from all over the country to support the first line of Hubei epidemic situation (5, 12).

At the same time, conflicts between doctors and patients in China are still difficult to resolve in recent years, mainly caused by the contradictions in the accumulation and distribution of medical resources, the defects of the medical system itself, and the low social trust between doctors and patients (13, 14). The total number of medical damage liability disputes was around 18,112 during 2019, almost 1.7 times of 2018 (15, 16). As The Lancet reported (17–19), Chinese doctors are under threat, which may explain the reason of the low professional happiness of Chinese doctors and the low conversion rate of Chinese medical students choosing to continue medical practice after graduation (13, 20–23). However, the relationship between doctors and patients seems to be more harmonious during this pandemic (24). Medical students in China, who cannot start their new term and are isolated at home, may be responding strongly to the COVID-19 pandemic, but no data are available to describe their perception and behaviors related to this infectious disease.

Generally, Chinese medical undergraduate education contains about 1-year public fundamental courses, nearly 2-year basic-medicine courses (physiology, pathology, anatomy, etc.), and 2-year clinical-medicine learning, including theoretical study (neurology, epidemiology, pediatrics, etc.) and clinical practice. The present study investigated the attitudes, knowledge, and social responsiveness of Chinese medical students in response to the pandemic for the first time, with the aim to compare the differences between medical students of different stages and better the phased cultivation of clinical medical students in the context of Chinese excellent doctor education program reform. Importantly, it is wise for us to put insight into public health and disease prevention, thus cultivating medical teams with enough public health knowledge in face of such major infectious events.

## Methods

### Development of the Questionnaire

The modified questionnaire, which was conducted in Chinese and derived from the seventh edition of diagnosis and treatment plan for pneumonia infected by novel coronavirus (25), was composed of three blocks as follows: (1) demographic characteristic of medical students, (2) attitudes and social responsiveness toward COVID-19 and the profession of doctors, and (3) knowledge related to COVID-19 including epidemiology and clinical manifestations.

The first section collected demographic data including age, gender, enrollment year, specialty choice, and whether he/she was the only child in his/her family. The second section reflecting the attitudes and social responsiveness contained eight questions, including five open-ended questions and three single-choice questions as follows: (1) “The main reasons for choosing medicine”; (2) “Supposing you were at work, your first choice during the pandemic would be”; (3) “Supposing your family is against you supporting Hubei, your choice would be”; (4) “The reasons for your choice of being a front-line worker in Wuhan”; (5) “Things I can do as a medical student during the pandemic”; (6) “Do you agree that doctors are full of happiness”; (7) “What is your future plan after COVID-19”; and (8) “Which clinical department will you work in”. Knowledge related to COVID-19 (the third section) was assessed by seven items, consisting of one single-choice question, and six multiple-choice questions where the respondent may only choose a single answer or may choose multiple answers.

### Sample Recruitment

This was the first study of clinical medical students' knowledge, attitudes, and social responsiveness toward COVID-19 in China. All participants were required to be medical students, whose specialty choice was Eight-year MD program or Five-year Undergraduate program in some Chinese well-known medical colleges, such as Xiangya Medical College of Central South University, West China School of Medicine of Sichuan University, and Peking University Health Science Center. The enrollment year of the participants was not used as an exclusion criterion in this study. Informed consents were obtained, and all questionnaires were administered anonymously between January 2020 and March 2020. All the participants in our study were enrolled *via* convenience sampling and were required to answer the questionnaire without any intervention by the external factors through an online platform named Wenjuanxing. Students with SARS-COV-2 infection were excluded from this study, which may affect the mental status of them. A total of 889 clinical medical students were enrolled in this study, including 428 students in Xiangya Medical College of Central South University, 244 students in West China School of Medicine of Sichuan University, and 217 students in Peking University Health Science Center.

### Statistical Analysis

Data from different universities were lumped together and were divided into different groups to compare the differences, including (1) students at the stage of learning clinical courses (Group A) or students at the basic-medicine stage (Group B) and (2) students who have graduated and worked (Group C) or newly enrolled (Group D). Data were expressed as the means and standard deviations (SDs) in the case of normally distributed data. Pearson's or Spearman's correlation tests were conducted to determine the correlations between variables. The associations between the independent variables and the dependent variables related to medical education were assessed by using univariate odds ratios (ORs) and their 95% confidence intervals (CIs). Statistical Product and Service Solutions version 26.0 (SPSS 26.0) was used for data analyses, with *p* < 0.05 as the level of statistical significance.

### Ethics

All participants signed an informed consent document as required by the institutional ethics committee. This study was approved by the ethics committees of the 3rd Xiangya Hospital of Central South University.

## Results

### Attitudes and Social Responsiveness Toward COVID-19 Among Chinese Clinical Medical Students

Table 1 shows the demographic characteristics of the participants, which matched the demography of the population to some extent (26–29). The proportion of students at different stages was balanced relatively (freshman, 26.3%; sophomore, 16.4%; junior student, 15.7%; senior student, 13.7%; fifth grade, 14.2%; senior 6 and above, 13.7%). It was found that 47.2% of the participants chose to volunteer to support Hubei Province and 46.9% chose to stick to their own hospitals if they had worked now (Table 2). Despite family opposition, 88.3% of the medical students surveyed were still willing to support the front-line in Wuhan, Hubei Province. Responsibilities of doctors were the main reason (92.7%) for those who wanted to work in the front-line (The vanguard and exemplary role of Communist Party members, 15.6%; Many doctors around me sign up for support, 8.2%; Be curious and want to experience, 11.4%; Others, 12.8%). As for the things medical students can do during the pandemic, the vast majority of participants said they would obey the arrangement of the government and the school (89.4%), encourage people around me to take active protective measures (86.3%), pay attention to the pandemic situation and learn efficiently (85.3%), and publish medical science articles and short videos (64.9%). When it came to the professional happiness of doctors, only 53% of the participants agreed or extraordinarily agreed Chinese doctors were full of happiness, 23.1% remained neutral, and 23.9% of the medical students were against the idea. In terms of target department, 12.5% of the participants preferred several departments with the heaviest workload during the outbreak (Department of infectious disease, 1.2%; Department of respiration, 3.6%; ICU or emergency, 7.7%), 87.5% preferred departments except abovementioned. The whole socio-demographic characteristics of the participants are presented in Table 2.

**Table 1:** Demographic characteristics of Chinese clinical medical students.

**Demographic characteristics**	***n***	**Percentage**	**Population (%)**
**Gender**			
Male	387	43.5	36–47
Female	502	56.5	
**Enrollment year**			
2019	234	26.3	–
2018	146	16.4	–
2017	140	15.7	–
2016	122	13.7	–
2015	126	14.2	–
2014 and before	121	13.7	–
**Specialty choice**			
Eight-year MD program	543	61.1	–
Five-year Undergraduate program	346	38.9	–
**The only child or not**			
Yes	556	62.5	45–50
No	333	37.5	

*The demography of the population was collected from the researches published in recent years, whose participants were clinical medical students in China (26–29)*.

**Table 2:** Attitudes and social responsiveness toward COVID-19 among Chinese clinical medical students.

**Questions**	***n***	**Percentage**
**The main reasons for choosing medicine**		
Dream and hobby	652	73.4
Medical family	78	8.8
Family recommendation	356	40.1
Stable job	340	38.3
High social status	158	17.8
Others	127	14.3
**Supposing you were at work, your first choice during the epidemic would be**		
Volunteer to support Hubei	410	47.2
Stick to your hospital	417	46.9
Ask for leave	12	1.3
Resign and change profession	4	0.4
Others	46	3.9
**Supposing your family is against you supporting Hubei, your choice would be**		
I will try my best to persuade my family and support Hubei	694	78.1
I will support Hubei without telling my family	91	10.2
I will follow my family's advice and not go to Hubei for support	104	11.7
**The reasons for your choice of being a front-line worker in Wuhan**		
Responsibilities of doctors	824	92.7
The vanguard and exemplary role of Communist Party members	139	15.6
Many doctors around me sign up for support	73	8.2
Be curious and want to experience	101	11.4
Others	114	12.8
**Things I can do as a medical student during the pandemic**		
Obey the arrangement of the government and the school	795	89.4
Encourage people around me to take active protective measures	767	86.3
Publish Medical science articles and short videos	577	64.9
Pay attention to the pandemic situation and learn efficiently	758	85.3
**Do you agree that doctors are full of happiness**		
Agree extraordinarily	101	11.4
Agree	370	41.6
Neutral	205	23.1
Disagree	167	18.8
Disagree extraordinarily	46	5.1
**What is your future plan after COVID-19**		
Clinical work in China	549	61.8
Clinical work abroad	29	3.3
Become a full-time scientific research personnel	24	2.7
Engaged in medical related profession	41	4.6
Not decided yet	231	26.0
Separated from the medical profession completely	2	0.2
Others	13	1.4
**Which clinical department will you work in**		
Department of infectious disease	11	1.2
Department of respiration	32	3.6
ICU or emergency	68	7.7
Departments except abovementioned	778	87.5

### Knowledge, Attitudes, and Social Responsiveness Toward COVID-19 in Medical Students at Different Learning Stages

#### Comparison of Knowledge of COVID-19 Between Students in Basic-Medicine and Clinical-Medicine Stages

A total of 302 students at the stage of learning clinical courses (Group A) and 426 students during the basic-medicine courses (Group B) were approached. Table 3 shows a similar percentage of gender, residence, and whether he/she was the only child in his/her family.

**Table 3:** Demographic characteristics of medical students in different groups.

**c**	**Group A *n* (%)**	**Group B *n* (%)**	***P*-value**	**Group C *n* (%)**	**Group D *n* (%)**	***P*-value**
N	302 (41.5)	426 (58.5)		75 (16.5)	380 (83.5)	
**Gender**						
Male	130 (43.1)	187 (43.8)	0.82	26 (34.7)	170 (44.7)	0.108
Female	172 (56.9)	239 (56.1)		49 (65.3)	210 (55.3)	
**Residence**						
Urban	236 (78.1)	325 (76.3)	0.558	52 (69.3)	298 (78.4)	0.088
Rural	66 (21.9)	101 (23.7)		23 (30.7)	82 (21.5)	
**The only child or not**						
Yes	191 (63.2)	264 (62.0)	0.727	36 (48.0)	256 (67.4)	**0.001**
No	111 (36.8)	162 (38.0)		39 (52.0)	124 (32.6)	

*p < 0.001 is indicated in bold*.

Table 4 shows medical students at the basic-medicine learning stage had a weaker knowledge toward COVID-19 than the clinical-medicine group, especially in clinical manifestations (*p* < 0.001). Less than 30% of the participants knew SARS-COV-2 is the correct name of the virus first-occurred in Wuhan, and nearly 70% confused the concepts of COVID-19 and SARS-COV-2. The significant differences in epidemiology between two groups were mainly reflected in the choices of chlorine-containing disinfectant (Group A: 73.1%, Group B: 61.5%, *p* < 0.01, OR: 1.71) and ultraviolet radiation (Group A: 66.5%, Group B: 55.2%, *p* < 0.01, OR: 1.62). Higher frequencies of right answer were found in group A among all the three questions related with clinical manifestations than group B. Besides, the bold OR indicated a significant association between better knowledge of COVID-19 and the clinical-medicine learning stage students.

**Table 4:** Knowledge related to COVID-19 including epidemiology and clinical manifestations.

**Question**	**Frequency of YES answer (%)**		**OR**
	**Group A *n* (%)**	**Group B *n* (%)**	
**Which one do you think is the name of the virus occurred first in Wuhan?**			
SARS-COV-2	84 (27.9)	112 (26.3)	1.08
COVID-19	203 (67.2)	279 (65.5)	1.08
MERSr-COV	14 (4.6)	25 (5.8)	0.78
Ebola virus^*^	1 (0.3)	10 (2.2)	**0.14**
**Which ways do you think are the distribution of SARS-COV-2?**^§^			
Droplet transmission	298 (98.7)	421 (98.8)	0.88
Air-borne transmission	166 (55.0)	236 (55.4)	0.98
Contagion^**^	247 (81.8)	306 (71.8)	1.76
Fecal–oral transmission	161 (53.3)	244 (57.3)	0.85
Mother–baby transmission	43 (14.2)	60 (14.1)	1.01
**Which masks do you think can obstruct SARS-COV-2?**^§^			
N95	302 (100.0)	424 (99.5)	/
PM2.5 respirator	30 (9.9)	37 (8.7)	1.16
Sponge mask	5 (1.6)	8 (1.9)	0.88
Active carbon mask	8 (2.6)	11 (2.6)	1.03
Surgical mask	290 (96.0)	412 (96.7)	0.82
**Which ways do you think can inactivate SARS-COV-2 effectively?**^§^			
Heating at 56°C for 30 min	270 (89.4)	383 (89.9)	0.95
75% ethyl alcohol	293 (97.0)	403 (94.6)	1.86
Chlorine-containing disinfectant^**^	221 (73.1)	262 (61.5)	1.71
Chlorhexidine	81 (26.8)	111 (26.1)	1.04
Ultraviolet radiation^**^	201 (66.5)	235 (55.2)	1.62
**What are the initial manifestations of COVID-19?**^§^			
Fever, weakness, and dry cough	301 (99.7)	424 (94.8)	1.42
Digestive symptoms, like nausea, vomiting, and diarrhea^***^	280 (92.7)	345 (81.0)	2.99
Neurological symptoms, such as headache^***^	187 (61.9)	201 (47.2)	1.82
Cardiovascular system symptoms, such as palpitation and chest tightness^**^	173 (57.3)	193 (45.3)	1.62
Ophthalmic symptoms, such as conjunctivitis^***^	220 (72.8)	193 (45.3)	**3.24**
Only mild limb or back muscle pain^***^	222 (72.9)	151 (35.4)	**5.05**
**Which of the following specimens can detect nucleic acids of SARS-COV-2?**^§^			
Nasopharyngeal swab^***^	290 (96.0)	343 (80.5)	**5.85**
Sputum^***^	279 (92.4)	311 (73.0)	**4.49**
Secretion of lower respiratory tract^***^	264 (87.4)	320 (75.1)	2.30
Blood	176 (58.3)	242 (56.8)	1.06
Feces^***^	253 (83.8)	291 (68.3)	2.40
**What are the criteria for the release of isolation and discharge of patients?**^§^			
Temperature returns to normal for more than 3 days^***^	252 (83.4)	298 (69.9)	2.16
Respiratory symptoms improved significantly^***^	223 (73.8)	248 (58.2)	2.03
Pulmonary imaging shows obvious absorption of inflammation^*^	245 (81.1)	318 (74.6)	1.46
The detection of respiratory pathogenic nucleic acid shows negative consecutive times (Sampling interval shall be at least 1 day)	300 (99.3)	411 (96.5)	**5.47**

*OR: odds ratio [(Medical students at clinical-learning stage confirmed/Medical students at clinical-learning stage not confirmed)/(Medical students at basic-medical learning stage confirmed/Medical students at basic-medical learning stage not confirmed)] OR>3 or OR < 1/3 is indicated in bold*.

§ *Multiple responses possible*.

* *p < 0.05*.

** *p < 0.01*.

*** *p < 0.001*.

#### Comparison of Knowledge, Attitudes, and Social Responsiveness Toward COVID-19 Between Graduated and Newly Enrolled Clinical Medical Students

**Figure 1:**
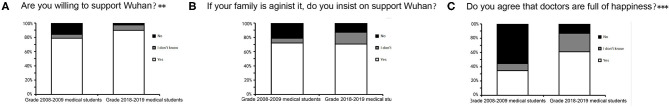
Questions that were considered to be reflected attitudes and social responsiveness. **(A)** Are you willing to support Wuhan? **(B)** If your family is against it, do you insist on supporting Wuhan? **(C)** Do you agree that doctors are full of happiness? ** *p*-value is < 0.01. ***p-value is < 0.001.

In this study, participants were grouped by whether they have graduated and worked or just entered school. Group C: Doctors enrolled in 2008 or 2009 when he/she was an undergraduate. Group D: Clinical medical students enrolled in 2018 or 2019. A total of 455 samples (Group C: 75; Group D: 380) were obtained, using the same questionnaire (Table 3). The percentage of totally correct answers in Group C was higher than that in Group D in most of the questions of epidemiology and clinical manifestations of COVID-19 (*p* < 0.001, Table 5). Interestingly, there were significant differences between the two groups in the attitudes of becoming a front-line doctor in Wuhan and the professional happiness of doctors. Further sub-analysis about the items above showed that Group D seemed more active than Group C (Figure 1). Surprisingly, the idea of supporting Wuhan diminished significantly after the objections of their family among newly enrolled medical students (Figures 1A,B).

**Table 5:** Percentage of totally correct answers to questions of COVID-19 between group C and group D.

**General knowledge of NPC section items**	**Totally correct answer (%)**		***P*-value**
	**Group C *n* (%)**	**Group D *n* (%)**	
Which one do you think is the name of the virus in Wuhan?^*^	22 (28.8)	72 (18.9)	0.042^*^
Which ways do you think are the distribution of SARS-COV-2?^***^	18 (24.0)	35 (9.1)	< 0.001^***^
Which masks do you think can obstruct SARS-COV-2?	67 (89.3)	335 (88.2)	0.772
Which ways do you think can inactivate SARS-COV-2 effectively?^***^	44 (58.7)	67 (17.6)	< 0.001^***^
What are the initial manifestations of COVID-19?^***^	35 (46.7)	52 (13.7)	< 0.001^***^
Which of the following specimens can detect nucleic acids of SARS-COV-2?^***^	38 (50.7)	92 (24.1)	< 0.001^***^
What are the criteria for the release of isolation and discharge of patients?^***^	55 (73.3)	166 (43.7)	< 0.001^***^

*p < 0.05 is indicated in bold*.

* *p < 0.05*,

** *p < 0.01*,

*** *p < 0.001*.

## Discussion

Medical education under the context of prevention and control of COVID-19 has been pushed to the forefront. It has gone beyond the scope of maintaining health and disease diagnosis and treatment and is increasingly linked to social responsiveness and national security, which we should attach vital importance to. In the face of new challenges and requirements to the pandemic, it is necessary to comprehensively analyze all kinds of problems that may occur in medical education reform and take precautions. Our study was one of the first hospital-based attempts to obtain an initial estimate of Chinese medical students' attitudes, knowledge, and social responsiveness toward COVID-19, especially under the impact of different learning stages of medical students, so as to provide effective suggestions on medical students' education.

Medical students during the clinical learning stage showed more solid knowledge toward COVID-19 than those of the basic-medicine stage, which was plausible by their deepening clinical knowledge and intellectual curiosity about disease (30). Intellectual curiosity, the core of the humanistic practice of medicine, is a desire for knowledge that leads to exploratory behavior, including an inherent and stable baseline trait (trait curiosity) and a variable context-dependent state (state curiosity) (31, 32). Studies in educational psychology suggested that trait curiosity is positively associated with academic achievement, and the educational process itself may influence the state curiosity of medical students (30, 33). What is more, for a long time, medical education in China has emphasized “treatment” more than “prevention.” Specifically, in the curriculum system of clinical, basic, and other majors in medical education, the proportion of public health courses is relatively small and the structure is unreasonable (34). There are few intersections between clinical and prevention teaching and few opportunities for students from clinical backgrounds to participate in public health practice. In addition, the curriculum setting of proofreading public health emergencies in most medical schools in China is far from enough, and the proportion of professional knowledge related to public health emergencies and psychological crisis management is seriously unbalanced, or even missing, and the contents are relatively outdated and lagging behind. The majority of medical personnel are obviously deficient in the knowledge and skills of responding to emergencies. They can only temporarily train and learn protective skills in the face of an outbreak, which increases the risk of infection (6, 35). In the early stage of the response to the epidemic, we had to reflect on the unexpected casualties caused by the insufficient public health literacy of front-line medical personnel. So we should strengthen the teaching management of public health and preventive medicine in medical majors. Moreover, the training of public health and epidemic prevention talents must be expanded in scale and improved in quality. Medical colleges must set up schools of public health and strengthen the construction of schools of public health. Only in this way can a team of professionals who not only know public health but also know systematic epidemic prevention and emergency response be trained quickly (36).

When it came to the attitudes and social responsiveness toward COVID-19, almost all participants chose to do a favor to the society as medical students or as doctors supposing they were at work, revealing great social cohesion and adaptability. However, opinions seemed to differ greatly when talking about the professional happiness of doctors, which was definitely related to the high ratio of medical disputes in China (37–39). Occupational well-being is the key to maintaining the quantity and quality of new entrants (40–42), which may explain the low transformation rate from medical students to doctors in China. Our results showed that newly enrolled medical students expected too much for professional happiness, which was much higher than that in the graduated group. Surprisingly, we found that the idea of newly enrolled medical students could be easily affected by interventions. In light of this information, more positive intervention such as policy guidance and communicative skills should be paid to medical students to maintain the high level of professional happiness, especially those newly enrolled. It is not only beneficial to medical students and doctors themselves but also conducive to ensuring the smooth and effective development of medical services and the stability and harmony of the entire society.

Being aware of the high conflict rate between doctors and patients in China, systemic managements and normalized media coverage and volunteerism have been performed to improve this situation (43–45). How to protect doctors from injuries in the process of practicing has always been the attention of the whole society. The Basic Health Care and Health Promotion Law is the first basic and comprehensive law in Chinese health field (45). The public have gradually realized the limitations of medicine during this pandemic, which will be helpful to build a stable foundation between the doctors and patients in China. Medical education is also constantly being reformed to cultivate modern high-quality doctors (46). Nowadays, medical education pays attention not only to the cultivation of professional knowledge and clinical skills but also to the comprehensive qualities including communicative abilities and empathy (47, 48). Surprisingly, a significant decline in empathy during medical school and residency was found in a systematic review, which was associated with gender, ethnicity, and specialty choice (49, 50). However, previous studies showed that well-received educational interventions could successfully cultivate empathy in undergraduate medical students, such as interventions of patient narrative and creative arts, writing, drama, communication skills training, and interpersonal skills training (51–53). It also emphasized the need for multicenter, randomized controlled trials, reporting long-term data to assess the longevity of intervention effects (51).

In addition, we further analyzed whether there were differences in knowledge and social responsiveness toward COVID-19 among participants of different demographic characteristics, because of the association of decreasing empathy and gender, ethnicity, and specialty choice (50). However, there were no significant differences in knowledge and social responsiveness toward COVID-19 among students in different gender (Male & Female), specialty choice (Eight-year MD program & Five-year Undergraduate program), and family (He/She is the only child & He/She is not the only child) (*p* both > 0.05).

However, there are some limitations in our study that must be acknowledged. (1) The participants may be worried about the confidentiality of this study since it was conducted by their peers, which may have impact on their responses. (2) We only focused on students in some well-known Chinese medical universities, which is a good attempt, but further research will need to be carried out to expand the context covering medical colleges at different levels so as to better match the demographic characteristics of the target population. Despite these limitations, our work provides a basis for international comparisons of medical students' knowledge, attitudes, and social responsiveness facing great public emergency health and safety problems.

## Conclusions

This is the first study to evaluate the knowledge, attitudes, and social responsiveness toward COVID-19 among Chinese medical students and found some differences between students at different stages to better the phased cultivation of clinical medical students during the medical education reform. Medical students during the clinical learning stage showed more solid knowledge toward COVID-19 than those of basic-medicine stage, probably due to the deepening clinical knowledge and intellectual curiosity about disease. When it came to the attitudes and social responsiveness toward COVID-19, almost all participants chose to do a favor to the society revealing great social cohesion and adaptability. Surprisingly, we found the idea of newly enrolled medical students could be easily affected by interventions. In light of this information, medical education should pay attention not only to the cultivation of professional knowledge and clinical skills but also to the positive interventions to cultivate the comprehensive qualities including communicative abilities and empathy.

## Data Availability Statement

The raw data supporting the conclusions of this article will be made available by the authors with requirement.

## Ethics Statement

The studies involving human participants were reviewed and approved by the ethics committee of the 3rd Xiangya Hospital of Central South University. The patients/participants provided their written informed consent to participate in this study.

## Author Contributions

The study idea was conceived by FW and HY. HY, YZ, and FW were responsible for the writing and revising manuscript. All authors were involved in the data collecting and statistics.

## Conflict of Interest

The authors declare that the research was conducted in the absence of any commercial or financial relationships that could be construed as a potential conflict of interest.
